# 2-Ferrocenyl-3-nitro-2*H*-chromene

**DOI:** 10.1107/S1600536813015730

**Published:** 2013-06-12

**Authors:** S. Paramasivam, Jonnalagadda Naga Siva Rao, P. R. Seshadri, Raghavachary Raghunathan

**Affiliations:** a Post Graduate and Research Department of Physics, Agurchand Manmull Jain College, Chennai 600 114, India; b Department of Organic Chemistry, University of Madras, Guindy Campus, Chennai 600 025, India

## Abstract

In the title compound, [Fe(C_5_H_5_)(C_14_H_10_NO_3_)], the cyclo­penta­dienyl rings are in an eclipsed conformation and the pyran ring adopts a half-chair conformation. The mean plane of the pyran ring makes dihedral angles of 79.33 (1) and 80.73 (1)°, respectively, with the substituted and unsubstituted cyclo­penta­dienyl rings. In the crystal, pairs of C—H⋯O hydrogen bonds link the mol­ecules into inversion dimers with *R*_2_^2^(16) motifs.

## Related literature

For the biological activity of ferrocenyl derivatives, see: Fouda *et al.* (2007 ▶); Jaouen *et al.* (2004 ▶); Biot *et al.* (2004 ▶); Edwards *et al.* (1975 ▶). For conformational analysis and puckering parameters, see: Cremer & Pople (1975 ▶). For related structures, see: Zora *et al.* (2006 ▶); Paramasivam *et al.* (2013 ▶). For graph-set notation, see: Bernstein *et al.* (1995 ▶).
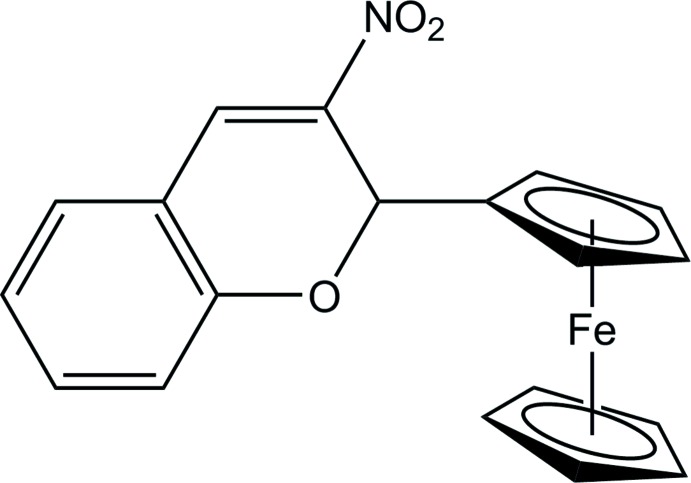


## Experimental

### 

#### Crystal data

[Fe(C_5_H_5_)(C_14_H_10_NO_3_)]
*M* *_r_* = 361.17
Monoclinic, 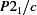

*a* = 10.047 (5) Å
*b* = 19.317 (5) Å
*c* = 8.135 (5) Å
β = 96.667 (5)°
*V* = 1568.1 (13) Å^3^
*Z* = 4
Mo *K*α radiation
μ = 0.98 mm^−1^
*T* = 298 K
0.30 × 0.25 × 0.20 mm

#### Data collection

Bruker SMART APEXII area-detector diffractometer
Absorption correction: multi-scan (*SADABS*; Bruker, 2008 ▶) *T*_min_ = 0.752, *T*_max_ = 0.822
14295 measured reflections
3878 independent reflections
3320 reflections with *I* > 2σ(*I*)
*R*_int_ = 0.032

#### Refinement

*R*[*F* ^2^ > 2σ(*F* ^2^)] = 0.028
*wR*(*F* ^2^) = 0.076
*S* = 1.01
3878 reflections
218 parameters
H-atom parameters constrained
Δρ_max_ = 0.27 e Å^−3^
Δρ_min_ = −0.27 e Å^−3^

### 

Data collection: *APEX2* (Bruker, 2008 ▶); cell refinement: *SAINT* (Bruker, 2008 ▶); data reduction: *SAINT*; program(s) used to solve structure: *SHELXS97* (Sheldrick, 2008 ▶); program(s) used to refine structure: *SHELXL97* (Sheldrick, 2008 ▶); molecular graphics: *ORTEP-3 for Windows* (Farrugia, 2012 ▶) and *PLATON* (Spek, 2009 ▶); software used to prepare material for publication: *SHELXL97*, *PLATON* (Spek, 2009 ▶) and *publCIF* (Westrip, 2010 ▶).

## Supplementary Material

Crystal structure: contains datablock(s) I, global. DOI: 10.1107/S1600536813015730/is5279sup1.cif

Structure factors: contains datablock(s) I. DOI: 10.1107/S1600536813015730/is5279Isup2.hkl

Additional supplementary materials:  crystallographic information; 3D view; checkCIF report

## Figures and Tables

**Table 1:** Hydrogen-bond geometry (Å, °)

*D*—H⋯*A*	*D*—H	H⋯*A*	*D*⋯*A*	*D*—H⋯*A*
C16—H16⋯O3^i^	0.93	2.55	3.390 (3)	150

Symmetry code: (i) 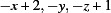.

**Fig. 1.**
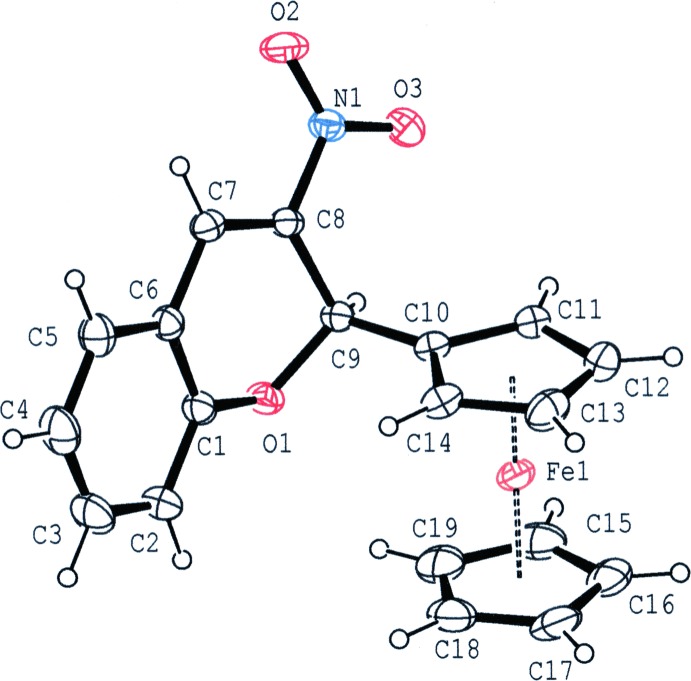
The molecular structure of the title compound, showing the atom-numbering scheme and displacement ellipsoids drawn at the 30% probability level.

**Fig. 2.**
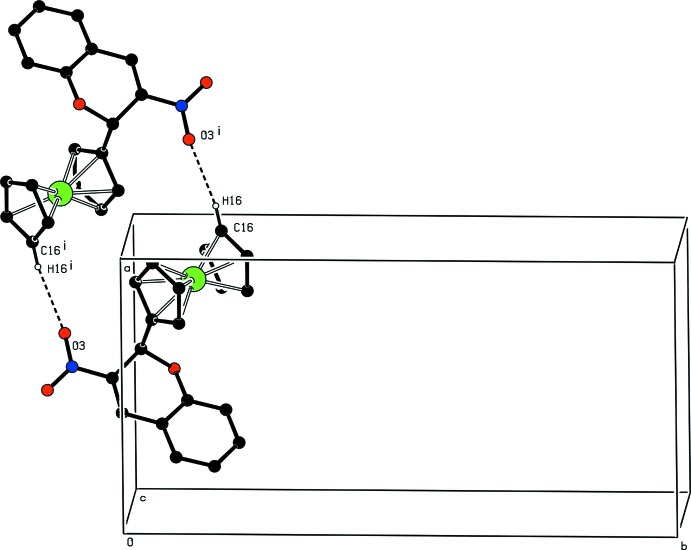
The packing arrangement of the title compound viewed down c axis, showing the formation of centrosymmetric R22(16) dimer. The dashed lines indicate C—H···O intermolecular interactions. H atoms not involved in hydrogen bonding have been omitted for clarity. [Symmetry code: (i) -x +2, -y, -z + 1.]

## supplementary crystallographic information

### Comment

As a continuation of our research related to ferrocenyl derivatives, we analyzed the crystal structure of (*E*)-1-ferrocenyl-3-[2-(2-hydroxyethoxy)phenyl]prop-2-en-1-one (Paramasivam *et al.*, 2013). The present compound exhibits the pronounced similarity to the previous ones, either in bond lengths and angles as well as in molecular conformation.

Ferrocenyl derivatives exhibit antibacterial (Fouda *et al.*, 2007), antitumor (Jaouen *et al.*, 2004), antifungal and antimalarial (Biot *et al.*, 2004) activities. It was proved that the replacement of the aromatic group by the ferrocenyl moiety in penicillins and cephalosporins could improve their antibiotic activity (Edwards *et al.*, 1975). Against this background, the title compound was chosen for X-ray structure analysis (Fig. 1).

In the title compound, the pyran ring (O1/C1/C6–C9) adopts a half-chair conformation with the puckering parameters (Cremer & Pople, 1975) being q2 = 0.304 (1) Å, q3 = -0.138 (1) Å, QT = 0.334 (1) Å and it makes dihedral angles of 79.66 (1) and 80.65 (1)°, respectively, with the substituted (C10–C14) and unsubstituted (C15–C19) cyclopentadienyl (Cp) rings of the ferrocenyl unit. In ferrocenyl unit, the two Cp rings are planar and are essentially parallel to each other with a dihedral angle of 2.08 (1)° between them. The Fe atom lies in the middle of the two planes of Cp rings. The distances of the Fe1 atom from the centroids of the substituted and unsubstituted cyclopentadienyl rings are 1.636 (13) and 1.646 (14) Å, respectively. The *Cg*1—Fe1—*Cg*2 angle is 178.09 (4)°, where *Cg*1 and *Cg*2 are the centroids of (C10–C14) and (C15–C19) Cp rings, respectively. The C—C bond distances in the Cp rings range from 1.403 (3) to 1.429 (2) Å, while Fe—C bond lengths range between 2.028 (1) and 2.040 (2) Å and all of which are as expected (Zora *et al.*, 2006). The torsion angles O1—C9—C10—C14 and C7—C8—N1—O2 [-42.8 (1)° and -6.1 (2)°, respectively] indicate the bent conformation of the molecule. The geometric parameters of the title compound (Fig. 1) agree well with the reported similar structures (Paramasivam *et al.*, 2013).

The crystal packing reveals a weak C—H···O hydrogen bonds (Fig. 2). In the crystal structure, the molecules at (-*x* + 2, -*y*, -*z* + 1) are linked by C16—H16···O3 hydrogen bond, generating a centrosymmetric dimeric ring motif *R*_2_^2^(16) (Bernstein *et al.*, 1995).

### Experimental

To a solution of salicylaldehyde (2 equiv) and (*E*)-(2-nitrovinyl) ferrocene (1 equiv), 1,4 diazabicyclo[2.2.2]octane (DABCO) (0.5 equiv) was added, and in the absence of solvent the reaction mixture was stirred at 45 °C for 2 h. After completion of the reaction as indicated by TLC, the crude product was extracted with ethylacetate, The organic layer was dried with anhydrous sodium sulfate and concentrated in *vacuo.* Then the crude product was purified by column chromatography using hexane/EtOAc (9:1) as eluent.

### Refinement

Hydrogen atoms were positioned geometrically and allowed to ride on their parent atoms, with C—H = 0.93–0.98 Å, and with *U*_iso_(H) = 1.2*U*_eq_(C).

### Crystal data

**Table:**

[Fe(C_5_H_5_)(C_14_H_10_NO_3_)]	*F*(000) = 744
*M**_r_* = 361.17	Monoclic
Monoclinic, *P*2_1_/*c*	*D*_x_ = 1.530 Mg m^−^^3^
Hall symbol: -P 2ybc	Mo *K*α radiation, λ = 0.71073 Å
*a* = 10.047 (5) Å	Cell parameters from 3878 reflections
*b* = 19.317 (5) Å	θ = 2.0–28.3°
*c* = 8.135 (5) Å	µ = 0.98 mm^−^^1^
β = 96.667 (5)°	*T* = 298 K
*V* = 1568.1 (13) Å^3^	Block, colourless
*Z* = 4	0.30 × 0.25 × 0.20 mm

### Data collection

**Table:**

Bruker SMART APEXII area-detector diffractometer	3878 independent reflections
Radiation source: fine-focus sealed tube	3320 reflections with *I* > 2σ(*I*)
Graphite monochromator	*R*_int_ = 0.032
ω and φ scans	θ_max_ = 28.3°, θ_min_ = 2.0°
Absorption correction: multi-scan (*SADABS*; Bruker, 2008)	*h* = −12→13
*T*_min_ = 0.752, *T*_max_ = 0.822	*k* = −25→25
14295 measured reflections	*l* = −10→10

### Refinement

**Table:**

Refinement on *F*^2^	Secondary atom site location: difference Fourier map
Least-squares matrix: full	Hydrogen site location: inferred from neighbouring sites
*R*[*F*^2^ > 2σ(*F*^2^)] = 0.028	H-atom parameters constrained
*wR*(*F*^2^) = 0.076	*w* = 1/[σ^2^(*F*_o_^2^) + (0.033*P*)^2^ + 0.4857*P*] where *P* = (*F*_o_^2^ + 2*F*_c_^2^)/3
*S* = 1.01	(Δ/σ)_max_ < 0.001
3878 reflections	Δρ_max_ = 0.27 e Å^−^^3^
218 parameters	Δρ_min_ = −0.27 e Å^−^^3^
0 restraints	Extinction correction: *SHELXL97* (Sheldrick, 2008), Fc^*^=kFc[1+0.001xFc^2^λ^3^/sin(2θ)]^-1/4^
Primary atom site location: structure-invariant direct methods	Extinction coefficient: 0.0231 (10)

### Special details

**Table:**

Geometry. All e.s.d.'s (except the e.s.d. in the dihedral angle between two l.s. planes) are estimated using the full covariance matrix. The cell e.s.d.'s are taken into account individually in the estimation of e.s.d.'s in distances, angles and torsion angles; correlations between e.s.d.'s in cell parameters are only used when they are defined by crystal symmetry. An approximate (isotropic) treatment of cell e.s.d.'s is used for estimating e.s.d.'s involving l.s. planes.
Refinement. Refinement of *F*^2^ against ALL reflections. The weighted *R*-factor *wR* and goodness of fit *S* are based on *F*^2^, conventional *R*-factors *R* are based on *F*, with *F* set to zero for negative *F*^2^. The threshold expression of *F*^2^ > σ(*F*^2^) is used only for calculating *R*-factors(gt) *etc*. and is not relevant to the choice of reflections for refinement. *R*-factors based on *F*^2^ are statistically about twice as large as those based on *F*, and *R*- factors based on ALL data will be even larger.

### Fractional atomic coordinates and isotropic or equivalent isotropic displacement parameters (Å^2^)

**Table:**

	*x*	*y*	*z*	*U*_iso_*/*U*_eq_	
Fe1	0.86764 (2)	0.122593 (11)	0.36654 (3)	0.03932 (9)	
O1	0.53463 (10)	0.08538 (5)	0.40784 (12)	0.0377 (2)	
O3	0.67181 (11)	−0.10978 (6)	0.34443 (16)	0.0499 (3)	
O2	0.48671 (13)	−0.13896 (6)	0.19888 (17)	0.0564 (3)	
C8	0.52228 (13)	−0.02353 (7)	0.26407 (16)	0.0310 (3)	
C7	0.41120 (13)	−0.00411 (7)	0.17099 (16)	0.0343 (3)	
H7	0.3591	−0.0364	0.1077	0.041*	
N1	0.56204 (12)	−0.09583 (6)	0.26937 (15)	0.0380 (3)	
C1	0.43906 (14)	0.11112 (7)	0.28951 (18)	0.0358 (3)	
C6	0.37266 (13)	0.06792 (7)	0.16975 (17)	0.0350 (3)	
C10	0.73280 (13)	0.04854 (7)	0.28505 (16)	0.0334 (3)	
C9	0.61296 (13)	0.02649 (7)	0.36567 (16)	0.0311 (3)	
H9	0.6460	0.0031	0.4693	0.037*	
C14	0.73990 (16)	0.10038 (8)	0.16104 (18)	0.0418 (3)	
H14	0.6695	0.1279	0.1142	0.050*	
C2	0.40451 (17)	0.18034 (9)	0.2994 (2)	0.0503 (4)	
H2	0.4481	0.2087	0.3809	0.060*	
C11	0.86294 (14)	0.01895 (8)	0.3209 (2)	0.0416 (3)	
H11	0.8869	−0.0161	0.3971	0.050*	
C5	0.27019 (15)	0.09517 (9)	0.0577 (2)	0.0459 (4)	
H5	0.2245	0.0668	−0.0223	0.055*	
C4	0.23676 (18)	0.16440 (10)	0.0658 (2)	0.0588 (5)	
H4	0.1691	0.1827	−0.0094	0.071*	
C13	0.87430 (18)	0.10230 (10)	0.1222 (2)	0.0540 (4)	
H13	0.9071	0.1315	0.0454	0.065*	
C3	0.3038 (2)	0.20631 (9)	0.1855 (3)	0.0612 (5)	
H3	0.2809	0.2529	0.1897	0.073*	
C16	1.02060 (19)	0.17053 (10)	0.5089 (3)	0.0621 (5)	
H16	1.1114	0.1609	0.5098	0.074*	
C19	0.8045 (2)	0.16867 (11)	0.5692 (3)	0.0644 (5)	
H19	0.7277	0.1575	0.6172	0.077*	
C12	0.94954 (16)	0.05243 (10)	0.2199 (2)	0.0538 (4)	
H12	1.0402	0.0431	0.2184	0.065*	
C15	0.9335 (2)	0.13943 (10)	0.6101 (2)	0.0625 (5)	
H15	0.9564	0.1056	0.6898	0.075*	
C18	0.8136 (2)	0.21766 (10)	0.4431 (3)	0.0651 (6)	
H18	0.7437	0.2446	0.3932	0.078*	
C17	0.9465 (2)	0.21885 (9)	0.4056 (3)	0.0650 (5)	
H17	0.9798	0.2466	0.3264	0.078*	

### Atomic displacement parameters (Å^2^)

**Table:**

	*U*^11^	*U*^22^	*U*^33^	*U*^12^	*U*^13^	*U*^23^
Fe1	0.03815 (13)	0.03059 (13)	0.04735 (14)	−0.00762 (8)	−0.00298 (9)	−0.00190 (9)
O1	0.0411 (5)	0.0328 (5)	0.0384 (5)	0.0026 (4)	0.0010 (4)	−0.0087 (4)
O3	0.0464 (6)	0.0370 (6)	0.0652 (7)	0.0085 (5)	0.0013 (5)	0.0051 (5)
O2	0.0640 (7)	0.0295 (6)	0.0730 (8)	−0.0069 (5)	−0.0030 (6)	−0.0103 (6)
C8	0.0352 (6)	0.0250 (6)	0.0334 (6)	−0.0023 (5)	0.0069 (5)	−0.0014 (5)
C7	0.0360 (6)	0.0327 (7)	0.0340 (6)	−0.0044 (5)	0.0037 (5)	−0.0034 (5)
N1	0.0439 (6)	0.0279 (6)	0.0430 (6)	0.0000 (5)	0.0084 (5)	−0.0001 (5)
C1	0.0362 (7)	0.0309 (7)	0.0415 (7)	0.0011 (5)	0.0093 (6)	0.0016 (6)
C6	0.0333 (6)	0.0347 (7)	0.0375 (7)	0.0009 (5)	0.0066 (5)	0.0027 (6)
C10	0.0364 (6)	0.0287 (7)	0.0345 (6)	−0.0050 (5)	0.0014 (5)	−0.0040 (5)
C9	0.0341 (6)	0.0265 (6)	0.0320 (6)	0.0002 (5)	0.0017 (5)	−0.0008 (5)
C14	0.0469 (8)	0.0400 (8)	0.0375 (7)	−0.0105 (6)	0.0002 (6)	0.0000 (6)
C2	0.0538 (9)	0.0317 (8)	0.0660 (10)	0.0031 (7)	0.0102 (8)	−0.0049 (7)
C11	0.0380 (7)	0.0324 (8)	0.0541 (8)	−0.0029 (6)	0.0036 (6)	−0.0054 (6)
C5	0.0397 (7)	0.0520 (10)	0.0455 (8)	0.0051 (7)	0.0032 (6)	0.0061 (7)
C4	0.0503 (9)	0.0566 (11)	0.0687 (11)	0.0174 (8)	0.0043 (8)	0.0200 (9)
C13	0.0576 (10)	0.0572 (11)	0.0495 (9)	−0.0189 (8)	0.0160 (8)	−0.0018 (8)
C3	0.0619 (10)	0.0364 (9)	0.0867 (13)	0.0153 (8)	0.0147 (10)	0.0118 (9)
C16	0.0506 (9)	0.0501 (11)	0.0791 (12)	−0.0092 (8)	−0.0194 (9)	−0.0113 (10)
C19	0.0669 (11)	0.0591 (12)	0.0667 (11)	−0.0139 (9)	0.0056 (9)	−0.0302 (10)
C12	0.0408 (8)	0.0514 (10)	0.0717 (11)	−0.0085 (7)	0.0167 (8)	−0.0104 (9)
C15	0.0843 (14)	0.0448 (10)	0.0527 (10)	−0.0026 (9)	−0.0159 (9)	−0.0089 (8)
C18	0.0631 (11)	0.0386 (9)	0.0864 (14)	0.0045 (8)	−0.0213 (10)	−0.0170 (10)
C17	0.0699 (12)	0.0370 (9)	0.0823 (13)	−0.0202 (8)	−0.0154 (10)	0.0009 (9)

### Geometric parameters (Å, º)

**Table:**

Fe1—C10	2.0280 (14)	C9—H9	0.9800
Fe1—C17	2.0315 (18)	C14—C13	1.423 (2)
Fe1—C14	2.0317 (17)	C14—H14	0.9300
Fe1—C18	2.0330 (19)	C2—C3	1.384 (3)
Fe1—C13	2.035 (2)	C2—H2	0.9300
Fe1—C11	2.0358 (16)	C11—C12	1.420 (2)
Fe1—C16	2.0364 (18)	C11—H11	0.9300
Fe1—C19	2.039 (2)	C5—C4	1.382 (3)
Fe1—C12	2.0407 (19)	C5—H5	0.9300
Fe1—C15	2.041 (2)	C4—C3	1.380 (3)
O1—C1	1.3715 (18)	C4—H4	0.9300
O1—C9	1.4471 (16)	C13—C12	1.411 (3)
O3—N1	1.2269 (17)	C13—H13	0.9300
O2—N1	1.2227 (17)	C3—H3	0.9300
C8—C7	1.328 (2)	C16—C15	1.404 (3)
C8—N1	1.4519 (18)	C16—C17	1.409 (3)
C8—C9	1.5072 (18)	C16—H16	0.9300
C7—C6	1.444 (2)	C19—C18	1.407 (3)
C7—H7	0.9300	C19—C15	1.417 (3)
C1—C2	1.386 (2)	C19—H19	0.9300
C1—C6	1.393 (2)	C12—H12	0.9300
C6—C5	1.397 (2)	C15—H15	0.9300
C10—C11	1.426 (2)	C18—C17	1.403 (3)
C10—C14	1.429 (2)	C18—H18	0.9300
C10—C9	1.4982 (19)	C17—H17	0.9300
			
C10—Fe1—C17	158.48 (7)	O1—C9—H9	107.4
C10—Fe1—C14	41.22 (6)	C10—C9—H9	107.4
C17—Fe1—C14	121.30 (7)	C8—C9—H9	107.4
C10—Fe1—C18	123.06 (7)	C13—C14—C10	107.55 (15)
C17—Fe1—C18	40.40 (9)	C13—C14—Fe1	69.64 (10)
C14—Fe1—C18	106.10 (8)	C10—C14—Fe1	69.25 (8)
C10—Fe1—C13	68.98 (6)	C13—C14—H14	126.2
C17—Fe1—C13	105.82 (8)	C10—C14—H14	126.2
C14—Fe1—C13	40.95 (7)	Fe1—C14—H14	126.5
C18—Fe1—C13	120.90 (9)	C3—C2—C1	118.63 (17)
C10—Fe1—C11	41.08 (6)	C3—C2—H2	120.7
C17—Fe1—C11	158.28 (8)	C1—C2—H2	120.7
C14—Fe1—C11	69.10 (7)	C12—C11—C10	107.93 (15)
C18—Fe1—C11	160.53 (8)	C12—C11—Fe1	69.80 (10)
C13—Fe1—C11	68.61 (7)	C10—C11—Fe1	69.17 (8)
C10—Fe1—C16	159.72 (7)	C12—C11—H11	126.0
C17—Fe1—C16	40.54 (8)	C10—C11—H11	126.0
C14—Fe1—C16	157.95 (8)	Fe1—C11—H11	126.6
C18—Fe1—C16	68.01 (8)	C4—C5—C6	119.88 (16)
C13—Fe1—C16	122.33 (9)	C4—C5—H5	120.1
C11—Fe1—C16	123.42 (7)	C6—C5—H5	120.1
C10—Fe1—C19	108.28 (7)	C3—C4—C5	119.99 (16)
C17—Fe1—C19	68.05 (9)	C3—C4—H4	120.0
C14—Fe1—C19	122.11 (8)	C5—C4—H4	120.0
C18—Fe1—C19	40.42 (9)	C12—C13—C14	108.49 (15)
C13—Fe1—C19	157.35 (9)	C12—C13—Fe1	69.96 (11)
C11—Fe1—C19	125.02 (8)	C14—C13—Fe1	69.41 (9)
C16—Fe1—C19	68.08 (9)	C12—C13—H13	125.8
C10—Fe1—C12	68.89 (7)	C14—C13—H13	125.8
C17—Fe1—C12	121.53 (9)	Fe1—C13—H13	126.5
C14—Fe1—C12	68.77 (8)	C4—C3—C2	121.23 (16)
C18—Fe1—C12	156.79 (9)	C4—C3—H3	119.4
C13—Fe1—C12	40.53 (8)	C2—C3—H3	119.4
C11—Fe1—C12	40.77 (7)	C15—C16—C17	108.10 (18)
C16—Fe1—C12	107.79 (8)	C15—C16—Fe1	70.02 (11)
C19—Fe1—C12	161.28 (9)	C17—C16—Fe1	69.54 (10)
C10—Fe1—C15	123.97 (8)	C15—C16—H16	126.0
C17—Fe1—C15	68.02 (9)	C17—C16—H16	126.0
C14—Fe1—C15	159.25 (8)	Fe1—C16—H16	126.1
C18—Fe1—C15	68.06 (8)	C18—C19—C15	107.68 (19)
C13—Fe1—C15	159.30 (9)	C18—C19—Fe1	69.57 (12)
C11—Fe1—C15	109.37 (7)	C15—C19—Fe1	69.75 (11)
C16—Fe1—C15	40.29 (9)	C18—C19—H19	126.2
C19—Fe1—C15	40.66 (9)	C15—C19—H19	126.2
C12—Fe1—C15	124.47 (9)	Fe1—C19—H19	126.1
C1—O1—C9	118.31 (11)	C13—C12—C11	108.23 (15)
C7—C8—N1	119.92 (12)	C13—C12—Fe1	69.51 (11)
C7—C8—C9	123.11 (13)	C11—C12—Fe1	69.43 (10)
N1—C8—C9	116.97 (11)	C13—C12—H12	125.9
C8—C7—C6	118.89 (13)	C11—C12—H12	125.9
C8—C7—H7	120.6	Fe1—C12—H12	126.8
C6—C7—H7	120.6	C16—C15—C19	107.89 (19)
O2—N1—O3	123.74 (13)	C16—C15—Fe1	69.68 (11)
O2—N1—C8	119.23 (13)	C19—C15—Fe1	69.60 (11)
O3—N1—C8	117.03 (12)	C16—C15—H15	126.1
O1—C1—C2	117.93 (14)	C19—C15—H15	126.1
O1—C1—C6	120.88 (13)	Fe1—C15—H15	126.2
C2—C1—C6	121.06 (14)	C17—C18—C19	108.30 (17)
C1—C6—C5	119.19 (14)	C17—C18—Fe1	69.75 (11)
C1—C6—C7	117.79 (12)	C19—C18—Fe1	70.02 (11)
C5—C6—C7	123.00 (14)	C17—C18—H18	125.8
C11—C10—C14	107.80 (13)	C19—C18—H18	125.8
C11—C10—C9	124.12 (13)	Fe1—C18—H18	126.0
C14—C10—C9	128.07 (13)	C18—C17—C16	108.03 (19)
C11—C10—Fe1	69.75 (8)	C18—C17—Fe1	69.86 (10)
C14—C10—Fe1	69.53 (8)	C16—C17—Fe1	69.92 (10)
C9—C10—Fe1	126.71 (10)	C18—C17—H17	126.0
O1—C9—C10	111.54 (11)	C16—C17—H17	126.0
O1—C9—C8	108.86 (11)	Fe1—C17—H17	125.8
C10—C9—C8	114.05 (11)		
			
N1—C8—C7—C6	179.06 (12)	C1—C2—C3—C4	1.2 (3)
C9—C8—C7—C6	−1.8 (2)	C10—Fe1—C16—C15	−45.6 (3)
C7—C8—N1—O2	−6.1 (2)	C17—Fe1—C16—C15	119.24 (19)
C9—C8—N1—O2	174.62 (13)	C14—Fe1—C16—C15	160.02 (18)
C7—C8—N1—O3	173.14 (13)	C18—Fe1—C16—C15	81.56 (14)
C9—C8—N1—O3	−6.09 (18)	C13—Fe1—C16—C15	−164.86 (12)
C9—O1—C1—C2	−153.62 (13)	C11—Fe1—C16—C15	−80.57 (14)
C9—O1—C1—C6	30.53 (18)	C19—Fe1—C16—C15	37.83 (13)
O1—C1—C6—C5	176.06 (13)	C12—Fe1—C16—C15	−122.74 (13)
C2—C1—C6—C5	0.3 (2)	C10—Fe1—C16—C17	−164.87 (19)
O1—C1—C6—C7	−2.15 (19)	C14—Fe1—C16—C17	40.8 (3)
C2—C1—C6—C7	−177.87 (14)	C18—Fe1—C16—C17	−37.67 (14)
C8—C7—C6—C1	−12.08 (19)	C13—Fe1—C16—C17	75.90 (16)
C8—C7—C6—C5	169.79 (14)	C11—Fe1—C16—C17	160.19 (13)
C17—Fe1—C10—C11	160.9 (2)	C19—Fe1—C16—C17	−81.40 (15)
C14—Fe1—C10—C11	119.05 (13)	C12—Fe1—C16—C17	118.03 (14)
C18—Fe1—C10—C11	−164.87 (11)	C15—Fe1—C16—C17	−119.24 (19)
C13—Fe1—C10—C11	81.15 (11)	C10—Fe1—C19—C18	−119.88 (11)
C16—Fe1—C10—C11	−46.7 (2)	C17—Fe1—C19—C18	37.51 (11)
C19—Fe1—C10—C11	−122.75 (11)	C14—Fe1—C19—C18	−76.59 (13)
C12—Fe1—C10—C11	37.58 (10)	C13—Fe1—C19—C18	−40.8 (2)
C15—Fe1—C10—C11	−80.55 (12)	C11—Fe1—C19—C18	−162.33 (10)
C17—Fe1—C10—C14	41.8 (2)	C16—Fe1—C19—C18	81.38 (12)
C18—Fe1—C10—C14	76.08 (12)	C12—Fe1—C19—C18	162.08 (19)
C13—Fe1—C10—C14	−37.90 (10)	C15—Fe1—C19—C18	118.88 (17)
C11—Fe1—C10—C14	−119.05 (13)	C10—Fe1—C19—C15	121.24 (12)
C16—Fe1—C10—C14	−165.7 (2)	C17—Fe1—C19—C15	−81.37 (13)
C19—Fe1—C10—C14	118.20 (11)	C14—Fe1—C19—C15	164.53 (11)
C12—Fe1—C10—C14	−81.47 (11)	C18—Fe1—C19—C15	−118.88 (17)
C15—Fe1—C10—C14	160.40 (11)	C13—Fe1—C19—C15	−159.69 (18)
C17—Fe1—C10—C9	−81.0 (3)	C11—Fe1—C19—C15	78.79 (14)
C14—Fe1—C10—C9	−122.87 (16)	C16—Fe1—C19—C15	−37.50 (12)
C18—Fe1—C10—C9	−46.79 (15)	C12—Fe1—C19—C15	43.2 (3)
C13—Fe1—C10—C9	−160.77 (14)	C14—C13—C12—C11	−0.1 (2)
C11—Fe1—C10—C9	118.08 (16)	Fe1—C13—C12—C11	58.80 (12)
C16—Fe1—C10—C9	71.4 (3)	C14—C13—C12—Fe1	−58.89 (12)
C19—Fe1—C10—C9	−4.67 (14)	C10—C11—C12—C13	−0.01 (19)
C12—Fe1—C10—C9	155.66 (14)	Fe1—C11—C12—C13	−58.85 (13)
C15—Fe1—C10—C9	37.53 (15)	C10—C11—C12—Fe1	58.85 (11)
C1—O1—C9—C10	86.39 (14)	C10—Fe1—C12—C13	81.94 (10)
C1—O1—C9—C8	−40.33 (15)	C17—Fe1—C12—C13	−76.98 (12)
C11—C10—C9—O1	137.94 (13)	C14—Fe1—C12—C13	37.58 (10)
C14—C10—C9—O1	−42.85 (18)	C18—Fe1—C12—C13	−43.7 (2)
Fe1—C10—C9—O1	48.87 (15)	C11—Fe1—C12—C13	119.80 (14)
C11—C10—C9—C8	−98.24 (16)	C16—Fe1—C12—C13	−119.29 (11)
C14—C10—C9—C8	80.98 (18)	C19—Fe1—C12—C13	166.7 (2)
Fe1—C10—C9—C8	172.69 (10)	C15—Fe1—C12—C13	−160.58 (11)
C7—C8—C9—O1	26.80 (17)	C10—Fe1—C12—C11	−37.85 (9)
N1—C8—C9—O1	−153.99 (11)	C17—Fe1—C12—C11	163.23 (10)
C7—C8—C9—C10	−98.46 (16)	C14—Fe1—C12—C11	−82.21 (10)
N1—C8—C9—C10	80.76 (15)	C18—Fe1—C12—C11	−163.54 (16)
C11—C10—C14—C13	−0.15 (17)	C13—Fe1—C12—C11	−119.80 (14)
C9—C10—C14—C13	−179.46 (14)	C16—Fe1—C12—C11	120.92 (11)
Fe1—C10—C14—C13	59.33 (11)	C19—Fe1—C12—C11	46.9 (3)
C11—C10—C14—Fe1	−59.48 (10)	C15—Fe1—C12—C11	79.63 (13)
C9—C10—C14—Fe1	121.21 (14)	C17—C16—C15—C19	0.0 (2)
C10—Fe1—C14—C13	−118.98 (15)	Fe1—C16—C15—C19	−59.33 (13)
C17—Fe1—C14—C13	77.66 (14)	C17—C16—C15—Fe1	59.33 (13)
C18—Fe1—C14—C13	118.88 (13)	C18—C19—C15—C16	−0.1 (2)
C11—Fe1—C14—C13	−81.04 (11)	Fe1—C19—C15—C16	59.38 (13)
C16—Fe1—C14—C13	47.9 (2)	C18—C19—C15—Fe1	−59.46 (13)
C19—Fe1—C14—C13	159.90 (12)	C10—Fe1—C15—C16	162.62 (11)
C12—Fe1—C14—C13	−37.20 (11)	C17—Fe1—C15—C16	−37.71 (12)
C15—Fe1—C14—C13	−170.7 (2)	C14—Fe1—C15—C16	−158.78 (19)
C17—Fe1—C14—C10	−163.36 (11)	C18—Fe1—C15—C16	−81.42 (13)
C18—Fe1—C14—C10	−122.14 (11)	C13—Fe1—C15—C16	38.6 (3)
C13—Fe1—C14—C10	118.98 (15)	C11—Fe1—C15—C16	119.22 (12)
C11—Fe1—C14—C10	37.95 (9)	C19—Fe1—C15—C16	−119.15 (18)
C16—Fe1—C14—C10	166.84 (18)	C12—Fe1—C15—C16	76.30 (14)
C19—Fe1—C14—C10	−81.11 (12)	C10—Fe1—C15—C19	−78.22 (14)
C12—Fe1—C14—C10	81.78 (10)	C17—Fe1—C15—C19	81.44 (14)
C15—Fe1—C14—C10	−51.7 (2)	C14—Fe1—C15—C19	−39.6 (3)
O1—C1—C2—C3	−177.03 (15)	C18—Fe1—C15—C19	37.73 (12)
C6—C1—C2—C3	−1.2 (2)	C13—Fe1—C15—C19	157.8 (2)
C14—C10—C11—C12	0.10 (17)	C11—Fe1—C15—C19	−121.63 (12)
C9—C10—C11—C12	179.45 (13)	C16—Fe1—C15—C19	119.15 (18)
Fe1—C10—C11—C12	−59.24 (11)	C12—Fe1—C15—C19	−164.54 (11)
C14—C10—C11—Fe1	59.34 (10)	C15—C19—C18—C17	0.1 (2)
C9—C10—C11—Fe1	−121.31 (13)	Fe1—C19—C18—C17	−59.45 (13)
C10—Fe1—C11—C12	119.40 (14)	C15—C19—C18—Fe1	59.57 (13)
C17—Fe1—C11—C12	−41.7 (2)	C10—Fe1—C18—C17	−161.43 (11)
C14—Fe1—C11—C12	81.33 (12)	C14—Fe1—C18—C17	−119.68 (12)
C18—Fe1—C11—C12	160.4 (2)	C13—Fe1—C18—C17	−77.70 (13)
C13—Fe1—C11—C12	37.27 (11)	C11—Fe1—C18—C17	167.60 (19)
C16—Fe1—C11—C12	−78.18 (14)	C16—Fe1—C18—C17	37.81 (13)
C19—Fe1—C11—C12	−163.38 (11)	C19—Fe1—C18—C17	119.36 (16)
C15—Fe1—C11—C12	−120.73 (12)	C12—Fe1—C18—C17	−46.1 (2)
C17—Fe1—C11—C10	−161.06 (19)	C15—Fe1—C18—C17	81.41 (13)
C14—Fe1—C11—C10	−38.07 (9)	C10—Fe1—C18—C19	79.21 (13)
C18—Fe1—C11—C10	41.0 (3)	C17—Fe1—C18—C19	−119.36 (16)
C13—Fe1—C11—C10	−82.13 (10)	C14—Fe1—C18—C19	120.96 (12)
C16—Fe1—C11—C10	162.42 (10)	C13—Fe1—C18—C19	162.94 (11)
C19—Fe1—C11—C10	77.22 (12)	C11—Fe1—C18—C19	48.2 (3)
C12—Fe1—C11—C10	−119.40 (14)	C16—Fe1—C18—C19	−81.56 (13)
C15—Fe1—C11—C10	119.87 (10)	C12—Fe1—C18—C19	−165.48 (17)
C1—C6—C5—C4	0.6 (2)	C15—Fe1—C18—C19	−37.95 (12)
C7—C6—C5—C4	178.67 (15)	C19—C18—C17—C16	−0.1 (2)
C6—C5—C4—C3	−0.6 (3)	Fe1—C18—C17—C16	−59.73 (13)
C10—C14—C13—C12	0.15 (19)	C19—C18—C17—Fe1	59.62 (13)
Fe1—C14—C13—C12	59.23 (13)	C15—C16—C17—C18	0.1 (2)
C10—C14—C13—Fe1	−59.08 (11)	Fe1—C16—C17—C18	59.70 (13)
C10—Fe1—C13—C12	−81.71 (10)	C15—C16—C17—Fe1	−59.63 (13)
C17—Fe1—C13—C12	120.33 (11)	C10—Fe1—C17—C18	46.7 (3)
C14—Fe1—C13—C12	−119.85 (14)	C14—Fe1—C17—C18	77.66 (15)
C18—Fe1—C13—C12	161.48 (10)	C13—Fe1—C17—C18	119.39 (13)
C11—Fe1—C13—C12	−37.49 (9)	C11—Fe1—C17—C18	−168.85 (18)
C16—Fe1—C13—C12	79.39 (12)	C16—Fe1—C17—C18	−119.02 (19)
C19—Fe1—C13—C12	−168.93 (18)	C19—Fe1—C17—C18	−37.53 (12)
C15—Fe1—C13—C12	50.9 (3)	C12—Fe1—C17—C18	160.53 (12)
C10—Fe1—C13—C14	38.14 (10)	C15—Fe1—C17—C18	−81.53 (14)
C17—Fe1—C13—C14	−119.83 (11)	C10—Fe1—C17—C16	165.72 (18)
C18—Fe1—C13—C14	−78.67 (12)	C14—Fe1—C17—C16	−163.32 (12)
C11—Fe1—C13—C14	82.35 (10)	C18—Fe1—C17—C16	119.02 (19)
C16—Fe1—C13—C14	−160.76 (10)	C13—Fe1—C17—C16	−121.60 (14)
C19—Fe1—C13—C14	−49.1 (2)	C11—Fe1—C17—C16	−49.8 (3)
C12—Fe1—C13—C14	119.85 (14)	C19—Fe1—C17—C16	81.49 (15)
C15—Fe1—C13—C14	170.70 (19)	C12—Fe1—C17—C16	−80.45 (16)
C5—C4—C3—C2	−0.3 (3)	C15—Fe1—C17—C16	37.49 (13)

### Hydrogen-bond geometry (Å, º)

**Table:**

*D*—H···*A*	*D*—H	H···*A*	*D*···*A*	*D*—H···*A*
C16—H16···O3^i^	0.93	2.55	3.390 (3)	150

Symmetry code: (i) −*x*+2, −*y*, −*z*+1.
